# Digital Health Innovation by Design: A Logic Model Scaffold for Rural, Regional, and Remote Settings

**DOI:** 10.3390/ijerph22111743

**Published:** 2025-11-18

**Authors:** Michelle A. Krahe, Nico Adams, Sarah L. Larkins

**Affiliations:** 1 College of Medicine and Dentistry, James Cook University, Douglas 4811, QLD, Australia; sarah.larkins@jcu.edu.au; 2 Northern Australian Regional Digital Health Collaborative, Douglas 4811, QLD, Australia; nico.adams@jcu.edu.au; 3 College of Science and Engineering, James Cook University, Douglas 4811, QLD, Australia

**Keywords:** digital health innovation, rural and remote health, logic model, implementation science, health systems design

## Abstract

Digital health innovations (DHIs) have the potential to transform access, continuity, and quality of healthcare in rural, regional, and remote (RRR) settings, yet they often fall short in practice. Barriers extend beyond infrastructure and technology to include workforce challenges and the complex realities of delivering care across diverse geographic, cultural, and social contexts. Effective DHIs must therefore be designed with local needs and systemic constraints in mind. Conventional logic models can align inputs and activities with intended outcomes, but their linear and static assumptions often fail to capture the adaptive, relational, and place-based nature of RRR health systems. This paper presents a logic model scaffold—an iterative, four-step process for planning, implementing, and evaluating DHIs in RRR settings. Informed by program theory and implementation science, the scaffold is illustrated through a case example from the Northern Australian Regional Digital Health Collaborative. The process involves understanding context and needs, aligning interventions with system enablers, translating these into targeted activities and outputs, and embedding reflexivity and iterative adaptation. Applying the scaffold from the earliest stages of planning enhances methodological rigor, transparency, and responsiveness to local priorities, workforce realities, and system-level enablers in RRR healthcare.

## 1. Introduction

Digital health innovations (DHIs) hold considerable promise to improving access, continuity, and quality of care in rural, regional, and remote (RRR) Australia. In practice, however, these benefits are often difficult to realize. Beyond infrastructure and technology, persistent workforce challenges—including professional isolation, shortages, and limited training opportunities—directly influence the feasibility, adoption, and sustainability of DHIs in these settings [1,2,3,4]. Addressing these challenges requires innovation designs that are explicitly aligned with workforce needs, local contexts, and system capabilities, ensuring that DHIs are both usable and impactful.

Rigorous DHIs are typically underpinned by theoretical frameworks, implemented with fidelity, and evaluated using evidence-informed approaches [5,6]. In RRR contexts, however, conventional notions of rigor—such as reliance on randomized controlled trials—are often impractical or ethically challenging, as service models must remain flexible and context-responsive [7,8,9]. Alternative approaches, including mixed methods, realist evaluation, and participatory designs, better capture contextual complexity, strengthen relevance, and build trust with diverse stakeholders [10,11,12,13]. These approaches emphasize not just whether DHIs work, but how, for whom, and under what circumstances [14,15]. Transparent reporting, attention to scalability and sustainability, and appropriate analytical methods further underpin rigor [16]. Despite these approaches, fragmented infrastructure, variable digital capability, and enduring inequities continue to constrain the design, implementation, and evaluation of DHIs [17,18]. Structured, practical tools that provide clarity, coherence, and contextual fit from the outset are therefore essential.

Logic models offer one such tool. By articulating relationships between inputs, activities, outputs, outcomes, and impacts, logic models provide a framework for planning, implementing, and evaluating innovations. They help surface assumptions, identify contextual factors, and clarify causal pathways, serving as a conceptual roadmap through complex systems [19,20,21,22]. Widely applied across health systems research, program evaluation, and implementation science [23,24,25,26], logic models strengthen intervention coherence, align components with system-level goals, and foster collaboration among diverse stakeholders. Increasingly, they are used as theories of change, informing both process and outcome evaluations, facilitating adaptation, and supporting the identification of enablers and barriers to success [27,28].

However, conventional logic models often fall short in RRR settings. Their linear assumptions rarely capture the dynamic, relational, and systemic pressures that define these contexts. Workforce shortages, systemic inequalities, and uneven digital maturity further complicate their application, risking oversimplification of complexity and constraining innovation. This gap highlights the need for approaches that retain the strengths of logic models while embedding relational, adaptive, and scalable elements to better support DHI implementation in RRR contexts. This paper responds to this need by presenting a case application of a four-step logic model scaffold, designed to strengthen planning, implementation, and evaluation of DHIs in RRR settings.

## 2. Materials and Methods

The Northern Australian Regional Digital Health Collaborative (NARDHC) was established in 2022 as a multi-stakeholder initiative supporting DHIs across Northern Australia (www.nardhc.org (accessed on 13 October 2025)). NARDHC brings together research institutions, health services, government agencies, technology developers, and community representatives to collaborate across five priority areas to:

1. Establish a dedicated initiative for digital innovation and investment, fostering strong research–industry collaborations.
2. Connect stakeholders across Northern Australia (industry partners, researchers, and local communities), through virtual technologies.
3. Support collaborative projects via seed funding and strategic guidance to drive digital health solutions.
4. Implement, evaluate, and disseminate innovations to promote the broad-scale adoption of effective digital health technologies.
5. Deliver skills-based training for students and the health workforce to develop capabilities in using digital health applications.

A case example of using the logic model scaffold described herein focuses on priority area five, illustrating how the scaffold can guide workforce capability development in practice. Rather than replacing the conventional logic model structure (Inputs → Activities → Outputs → Outcomes → Impact), the scaffold wraps around the logic model, strengthening both its construction and application in RRR contexts (Table 1). The scaffold’s novelty lies in its explicit integration of local context, system-level enablers, and relational considerations, ensuring that all elements of the logic model are aligned with the complex realities of RRR health systems. While conventional logic models provide a static roadmap for planning and evaluation, the scaffold embeds iterative refinement, reflexive practice, and stakeholder co-design as core components. This approach adds unique value for DHI design in RRR settings by operationalizing context (mapping inputs, activities, and outcomes to geographic, cultural, and workforce realities), enhancing adaptivity (enabling continuous revision in response to emerging challenges, workforce changes, or policy shifts), and embedding relationality (systematically incorporating power dynamics, stakeholder perspectives, and co-design feedback). By foregrounding these dimensions, the logic model scaffold complements existing models while providing a practical, context-sensitive, and scalable framework for planning, implementing, and evaluating DHIs in complex RRR health systems. Table 1 illustrates these key distinctions and the complementary value added by the scaffold relative to conventional logic models.

The logic model scaffold was informed by established resources in program theory and evaluation, including the University of Wisconsin Logic Model training [29], the W. K. Kellogg Foundation Logic Model Development Guide [19], the CDC Evaluation Framework, and guidance from the Australian Institute of Family Studies on planning and evaluation [30]. These resources provided the theoretical grounding and practical scaffolding needed to design a fit-for-purpose framework. Development followed an iterative and consultative process that combined evidence review with stakeholder engagement. Key digital health capability frameworks, implementation science literature, evaluation reports, and national and regional workforce strategies were reviewed to identify existing principles and gaps [31,32,33]. This was complemented by consultation with NARDHC’s cross-sector network, including researchers, public health and primary care services, government and innovation agencies, and industry technology partners.

**Figure 1:**
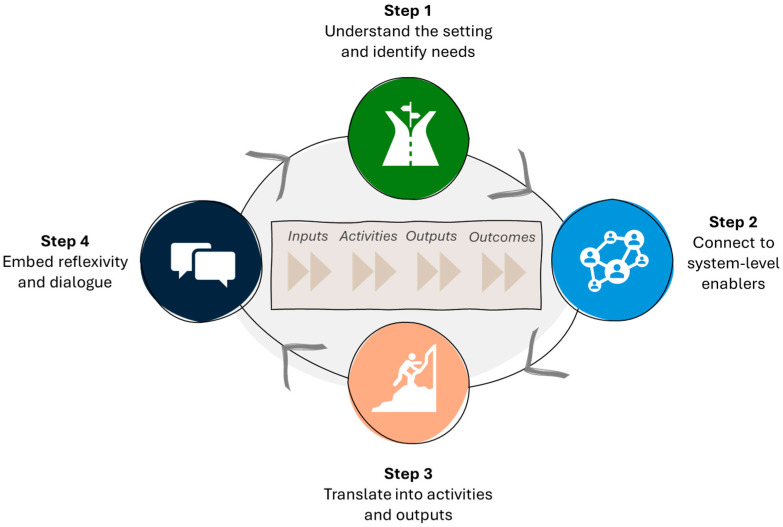
Steps in the logic model scaffold.

A “starting with the end in mind” approach, informed by backward mapping techniques from implementation science [29], was applied to anchor the scaffold in long-term outcomes. From these outcomes, the team worked backwards to identify the intermediate conditions, strategies, and resources required to achieve them. This ensured that the scaffold was outcomes-driven, contextually grounded, and aligned to RRR realities from the outset. Each step contributed a distinct layer of insight: Steps 1–2 grounded the process in local context and system enablers, Step 3 translated these insights into concrete activities and outputs, and Step 4 embedded reflexivity and relationality to ensure ongoing adaptation. Together, these steps provided a rigorous yet practical approach that complement a conventional logic model when developing DHIs in RRR settings (Figure 1).

### 2.1. Step 1: Understand the Setting and Identify Needs

This step focused on grounding understanding of the context in which a DHI will operate. RRR health systems are shaped by distinctive geographic, social, cultural, and structural conditions, including higher rates of hospitalizations, deaths, and injuries, as well as poorer access to and use of primary healthcare services compared with major cities [34]. Mapping this context involved identifying population distribution, service availability, workforce structures, and infrastructure, while also recognizing how these resources had been accessed and experienced by communities.

Place-based enablers (e.g., trusted relationships, community-led models) and barriers (e.g., digital exclusion, high mobility, infrastructure gaps) were identified early [2,35]. Diverse stakeholders including community members, service providers, clinicians, and policymakers were engaged to articulate a shared purpose using participatory methods such as co-design workshops, yarning circles, interviews, and collaborative planning sessions [36]. This step ensured priorities reflected stakeholder views, extending beyond technical outcomes to conditions that support digital health sustainability in RRR settings.

### 2.2. Step 2: Connect to System-Level Enablers

Sustainability and scalability required alignment with system-level enablers across organizational, policy, and infrastructural levels. This scaffold integrated established theoretical approaches, including implementation science, systems thinking, and behavior change models, to clarify mechanisms of action and enhance its utility for planning, implementation, and evaluation [8,37]. During this step, assumptions about scalability were assessed, and key dependencies were identified. Behavior change frameworks, including the Capability, Opportunity, Motivation and Behavior (COM-B) model and the Theoretical Domains Framework (TDF), were used to map barriers to targeted intervention components, ensuring that training addresses capability, opportunity, and motivation. Engagement with policymakers, funders, and system leaders ensured alignment with existing health structures, place-sensitive investment, and governance arrangements.

### 2.3. Step 3: Translate into Activities and Outputs

Service delivery in RRR contexts is dynamic, adaptive, and shaped by workforce, community, and system-level factors. Using insights from Steps 1 and 2, the scaffold guided the translation of context and system enablers into specific activities, inputs, and outputs within a traditional logic model structure. Iterative reflection, stakeholder feedback, and adaptive learning were used to refine these components. The outcome was a logic model that was both practical and responsive, supporting implementation, evaluation, and progressive workforce capability development.

### 2.4. Step 4: Embed Reflexivity and Dialogue

The success of a DHI in RRR contexts depends not only on technical design and response to need, but also on how well it adapts to the complex human, cultural, and relational dynamics at play. Reflexivity (critical self-reflection) and relationality (attention to relationships and power) were central to this step. Stakeholders, including clinicians, community members, service providers, policymakers, and technology partners engaged in ongoing dialogue to reflect on assumptions, expectations, and experiences. Power imbalances, cultural identities, and institutional structures shaped trust, participation, and decision-making [8,37]. The scaffold was treated as a living document, revisited and revised regularly to integrate new insights and evolving relationships. This ensured interventions remain relevant, culturally safe, and responsive to local realities, producing a dynamic, evolving set of inputs, activities, outputs, and outcomes.

## 3. Results

This section presents the application of the logic model scaffold during the design stage of the NARDHC digital health capability initiative.

### 3.1. Context Profile and Workforce Priorities

Healthcare delivery in northern Australia’s RRR regions is shaped by complex geographic, social, and systemic conditions, including geographic isolation, variable infrastructure, limited workforce capacity, and inequities in access to care [38]. These challenges contribute to uneven adoption of digital health technologies and variable integration into local systems. Access to training and professional development is often constrained, further limiting workforce readiness.

Grounding the scaffold in local context involved engaging 25 stakeholders, including community members, clinicians, service providers, and policymakers, to understand their experiences, priorities, and challenges. Stakeholders contributed through collaborative planning sessions. Data collected included meeting summaries and reflective logs, which were analyzed thematically to identify patterns, enablers, and barriers relevant to digital health implementation in RRR settings. Workforce-centered priorities extended beyond technical skills to include confidence building, cultural safety, alignment with professional identity, reduction in digital workload, and integration into local workflows. Place-based enablers (e.g., trusted relationships, community-led models) and barriers (e.g., digital exclusion, high mobility, infrastructure gaps) were explicitly mapped.

Step 1 produced a context profile and a set of workforce priorities that informed subsequent design, implementation, and evaluation of the digital health capability program (Box 1). By articulating workforce needs and desired outcomes upfront, this step ensured the initiative was responsive to RRR realities while aligned with broader objectives of equity, workforce retention, and service quality. The iterative analysis and synthesis of stakeholder input also strengthened transparency, reproducibility, and alignment with local and system-level enablers.

Box 1 **Context profile for the digital health capability initiative (Step 1).**

Place:

Northern Australia’s RRR health workforce faces geographic isolation, workforce shortages, limited digital infrastructure, cultural and linguistic diversity, and constrained access to training. These factors hinder access to care and the effective adoption and integration of digital health tools.

Purpose:

To build a confident and capable RRR health workforce that can sustainably use digital health innovations, thereby strengthening service delivery and access, equity, and system responsiveness.

Needs:

- Tailored, relevant, and context-sensitive digital health training.
- Opportunities for skills development, mentoring, and ongoing support.
- Align with local models of care, policy frameworks, and system priorities.

Goals:

- Equip RRR health workforce with digital capabilities needed to integrate DHIs into routine practice.
- Enhance workforce confidence, engagement, and retention.
- Reduce regional digital inequities and improve service quality and accessibility.

### 3.2. Mapping Workforce Needs to System-Level Enablers

Sustainability and scalability required linking workforce priorities with broader organizational, policy, and infrastructural enablers. The scaffold integrated established theoretical approaches (e.g., implementation science, systems thinking, or behavior change models) to clarify mechanisms of action and enhance its utility for planning and evaluation [8,37]. Critical dependencies such as digital system interoperability, availability of training infrastructure, leadership support, and secure funding streams were identified. Potential risks, including digital exclusion (for patients and healthcare providers), staff burnout, and unintended inequities, were explicitly considered to inform mitigation strategies.

A mapping process operationalized the link between workforce capability needs and system-level enablers, involving:

1. Mapping workforce barriers and priorities identified in Step 1 against relevant behavioral determinants using the COM-B model and the TDF.
2. Aligning each barrier with program responses (e.g., training modules, peer support, credentialing) and corresponding mechanisms of action.
3. Validating the map with NARDHC stakeholders, including health services, policymakers, and educators, to ensure coherence with local governance arrangements and system priorities.

The output of this step explicitly linked workforce barriers, behavioral determinants, program responses, and mechanisms of action (Table 2). This table provided a practical planning tool, ensuring that capability-building activities were tailored to RRR workforce needs and positioned for integration, scalability, and sustainability within broader health system structures.

### 3.3. Designing Activities and Outputs

Workforce needs and system-level enablers were operationalized into targeted, contextually grounded activities, including:

- Skills-based training modules reflecting real-world RRR care scenarios, aligned to national frameworks.
- Self-assessment tools, scenario-based learning, and credentialing mechanisms such as digital badges and CPD recognition.
- Virtual communities of practice, peer mentoring, and interactive learning sessions to support collaboration and continuous learning.
- Learning content tailored to the formal care workforce segments.
- Dashboards, feedback loops, and communication campaigns to monitor uptake, engagement, and learning outcomes.

Together, these activities produced a conventional logic model (Table 3) linking inputs, activities, and outputs to measurable outcomes, supporting adaptive implementation, iterative refinement, and evaluation in RRR settings.

### 3.4. Reflexivity and Iterative Refinement

The success of DHIs in RRR contexts depends not only on technical design as well as adaption to complex human, cultural, and relational dynamics. Reflexivity (critical self-reflection) and relationality (attention to relationships and power) were central to this process. The scaffold and logic model were treated as living documents, revisited regularly to integrate new insights and evolving relationships. Key outputs of this iterative process included:

- Updated versions of the logic model reflecting new learning and stakeholder feedback.
- Records of stakeholder dialogue (e.g., meeting summaries, reflective notes, or feedback reports) capturing evolving priorities, assumptions, and relational dynamics.
- Actionable refinements to intervention activities, evaluation measures, or governance processes emerging from reflexive reflection.

These outputs ensured interventions remained relevant, culturally safe, and responsive to local realities, producing a dynamic, evolving set of inputs, activities, outputs, and outcomes. By embedding context, system-level enablers, and reflexive processes within a traditional logic model, the scaffold functions as a practical tool for guiding implementation, aligning stakeholders, and supporting ongoing evaluation, addressing feasibility, adoption, and sustainability challenges unique to RRR health systems.

## 4. Discussion

This paper illustrates the value of a logic model scaffold in enhancing the planning, implementation, and evaluation of DHIs in RRR settings. Using the NARDHC digital health capability initiative as an example, we demonstrate how the logic model scaffold provides a systematic yet flexible structure for supporting DHI development. By explicitly embedding local context, system-level enablers, and reflexive processes, the scaffold moves beyond conventional logic models to support adaptive, context-sensitive, and sustainable interventions in complex RRR health systems.

RRR health systems are shaped by complex and dynamic challenges, including persistent workforce shortages, service fragmentation, and entrenched inequities in access to care [44]. In these settings, DHIs hold significant potential to improve service access, care coordination, and workforce sustainability. However, realizing these benefits requires a health workforce that is confident, capable, and supported to use digital tools effectively within contextually complex environments.

The logic model scaffold was developed as a living, adaptive tool to guide intervention planning, articulating how targeted, skills-based, contextually relevant training can contribute to workforce development. It provides a structured approach of mapping intervention goals, resources, planned activities, and anticipated outcomes while remaining responsive to local realities. This aligns with prior research highlighting the value of logic models in clarifying causal pathways, surfacing assumptions, and strengthening alignment between intervention design and intended impact [23]. In workforce development, such tools are particularly useful because they make explicit the mechanisms through which training initiatives are expected to build capability, increase confidence, and improve application of digital health tools in practice. By capturing variability in digital readiness, infrastructure, and workforce conditions, the scaffold helps align diverse stakeholders around shared objectives and identifies key enablers and barriers early in the process [5,45].

Implementation required significant investment of time and resources, particularly for collaborating with stakeholders to ensure contextual relevance and workforce alignment. Yet this process strengthens credibility, practical utility, and adaptability. In RRR contexts, where governance is fragmented, collaboration is essential, and policy landscapes shift frequently, so co-designed logic models support adaptive delivery by embedding reflexivity and responsiveness. They encourage inclusive dialogue, foster shared ownership, and generate actionable insights that allow DHIs to remain relevant in dynamic conditions, reducing the risk of failure due to rigid design.

Evaluation in real-world RRR healthcare systems is inherently challenging, as traditional methods often overlook socio-cultural, organizational, and contextual influences on how DHIs are implemented and experienced [46]. Logic models provide a transparent mechanism for mapping assumptions and tracing the pathways through which activities are expected to lead to changes in knowledge, behaviors, and system-level outcomes [26]. The scaffold strengthens evaluation by embedding place-based challenges, workforce needs, cultural considerations, and system-level enablers from the outset. By visualizing incremental progress alongside broader system-level goals such as retention, equity, and sustainability, the scaffold enables nuanced, ongoing assessment. Its iterative design ensures evolution in response to new learning, emerging challenges, and changes in service or policy contexts, aligning with continuous quality improvement and implementation science principles [47]. While existing implementation models provide a rigorous framework for mapping determinants, strategies, mechanisms, and outcomes [26], the scaffold complements these approaches by foregrounding the realities of RRR health settings. Step 5 emphasizes reflexivity and scalability, bridging practical implementation planning with formal implementation research. In doing so, the scaffold supports adaptive, relational, and outcomes-driven program design while offering a practical framework for evaluating impact across multiple levels of the health system.

Although developed for RRR Australian health systems, the scaffold’s principles may be applicable to other settings, including low- and middle-income countries (LMICs) or urban health systems facing complex service delivery challenges. Features such as iterative adaptation, reflexive engagement, and integration of system-level enablers are broadly relevant where workforce constraints, service fragmentation, or socio-cultural diversity influence implementation. However, applying the scaffold in new settings may require careful tailoring. For example, LMIC contexts may face infrastructure limitations, differing governance structures, or resource constraints, necessitating simplification or prioritization of scaffold elements. Urban systems may require adaptation to larger stakeholder networks and rapidly changing service landscapes. These considerations highlight the importance of context-sensitive adaptation while preserving the scaffold’s core principles.

This manuscript primarily presents a conceptual and methodological framework, contributing a structured, context-sensitive, practical approach for planning, implementing, and evaluating DHIs in RRR settings. The scaffold explicitly integrates local context, system-level enablers, iterative refinement, and stakeholder engagement—dimensions often underrepresented in conventional logic models. Although formal causal testing and generalizability are beyond this paper’s scope, the NARDHC case illustrates the scaffold’s practical application, and future work will empirically assess effectiveness, mechanisms of action, and adaptability across diverse health systems. This approach ensures the scaffold is principled yet adaptable, providing a foundation for evidence-informed DHI implementation while recognizing the importance of context-specific tailoring.

### 4.1. Limitations and Future Directions

The scaffold provides a structured, evidence-informed framework, but its effectiveness depends on the quality of planning, implementation, and evaluation. Collaboration requires time and resources, which can be challenging in dispersed RRR regions. Future work should explore scalable strategies for engaging diverse stakeholders early, particularly in dispersed regions where participation is logistically challenging. Implementation success depends on partnerships, organizational commitment, and the willingness of individuals to adopt and apply digital health capability frameworks. Research should examine how governance arrangements, funding structures, and cross-sector relationships shape adoption, adaptation, and sustainability of the model in practice. While the scaffold provides a transparency for evaluation, further work is needed to strengthen the evidence base on causal mechanisms and long-term outcomes. Although situating the scaffold alongside international digital health frameworks may offer additional insights, this comparison is beyond the scope of the current study. A review of such frameworks is underway, and findings will inform future research. Future studies should also explore longitudinal impacts of digital health capability-building on workforce behaviors, service delivery models, and health outcomes in RRR settings. Additionally, while the scaffold focuses on healthcare professionals and students, subsequent iterations should incorporate consumers and community perspectives, reflecting the increasing role of digital tools in patient self-management and care navigation.

### 4.2. Implications for Practice

This study reinforces the importance of grounding DHIs within a clear program theory that articulates the relationships between context, inputs, activities, and intended outcomes. The scaffold’s practical utility and potential benefits are illustrated through the NARDHC case; formal empirical evaluation of outcomes will be addressed in future research. Logic models enhance coherence, rigor, and transparency by clarifying assumptions, identifying enabling conditions, and supporting alignment across stakeholders. In RRR settings where infrastructure and workforce capacity vary, logic models offer practical tools for navigating complexity, aligning resources, and embedding adaptive learning cycles throughout implementation. They ensure capability-building efforts remain responsive to local needs, grounded in evidence, and positioned to deliver tangible benefits for health professionals, services, and communities. For policymakers and system leaders, embedding logic models within commissioning processes, digital health strategies, and program funding decisions supports accountability by ensuring initiatives have clear rationales, realistic pathways to impact, and a commitment to learning and adaptation. In RRR contexts, this approach targets investment towards initiatives that align with workforce realities, address persistent capability gaps, and strengthen equitable, sustainable digital health integration.

## 5. Conclusions

The logic model scaffold provides a structured yet flexible framework for planning, implementing, and evaluating DHIs in RRR settings. By grounding interventions in local context, engaging stakeholders, and aligning with system enablers, the scaffold supports effective planning of workforce capability initiatives, enables adaptive and reflective implementation, and facilitates robust evaluation of outcomes and impact. Applying a logic model from the earliest stages of DHI planning can strengthen methodological rigor, enhances transparency, and ensures innovations remain responsive to local priorities, workforce realities, and system-level enablers in RRR contexts.

## Figures and Tables

**Table 1:** Key differences between the logic model scaffold and conventional logic models.

Feature	Logic Model Scaffold	Conventional Logic Model	Complementary Value
Primary purpose	Guides planning, implementation, and evaluation of DHIs in RRR settings	Supports program planning, implementation, and evaluation in a variety of settings	Enhances conventional logic models with RRR-specific relevance
Focus on context	Central to all steps in the scaffold: context shapes inputs, activities, and outcomes	Often treated as background or assumptions	Ensures that inputs, activities, and outcomes are aligned to the realities of RRR health systems
Structure	Wraps around a conventional logic model with iterative scaffolding, co-design, and system alignment	Typically linear and static	Captures adaptive, relational, and systems-informed aspects of complex health systems
Stakeholder engagement & reflexivity	Embedded in steps 1–4: includes continuous feedback, power dynamics, co-design, and relational considerations	May be included, but often optional and not structured	Embedding stakeholder input ensures the model reflects lived experiences and system realities, improving relevance and feasibility
Adaptivity & iteration	Designed for iterative refinement, responsive to changing context, workforce needs, and emerging challenges	Limited adaptation, static snapshot of planned activities and outcomes	Provides a mechanism to revise and refine the model as context, system, and workforce needs evolve

**Table 2:** Barriers and targeted responses for building RRR digital health capability (Step 2).

Barrier	Why It Matters?	Behavior ^a^	Response	Mechanism of Action
Limited digital health capability [31,39]	Without foundational knowledge and skills, professionals, and consumers in RRR areas cannot effectively use digital tools, limiting quality and coordination of care	Knowledge, Skills → Capability	Type: Training and education; Mode: Online, embedded in curricula; Content: Skills-based modules using RRR scenarios; micro-credentials aligned to national frameworks	Builds core digital literacy; recognition through micro-credentials enhances motivation and value
Low confidence and engagement [5,40]	Even when training is available, many lack confidence or motivation to engage, particularly if content feels irrelevant or inaccessible	Beliefs about capabilities, Optimism, Reinforcement → Motivation	Type: Motivational and reflective learning; Mode: Online, credentialed learning; Content: Self-assessments, RRR-based scenarios, CPD recognition	Strengthens self-efficacy through relatable examples; rewards validated learning and sustains participation.
Workforce isolation [2,41]	Isolation limits peer learning, knowledge-sharing, and professional growth opportunities	Social influences, Role identity → Opportunity, Motivation	Type: Social learning and networking; Mode: Virtual peer groups, digital platforms; Content: Communities of practice, mentoring, local champions	Promotes shared learning, peer support, and identity as digital health leaders
Poor alignment with local practice [33,42]	Training that ignores RRR contexts disengages learners and risks poor adoption	Environmental context, Behavioral regulation → Opportunity, Capability	Type: Co-design and contextual learning; Mode: Stakeholder workshops, modular delivery; Content: Co-designed training, role-specific modules, culturally relevant case studies	Improves relevance and usability; co-design increases engagement and application in practice
Limited system support [43]	Weak infrastructure or promotion reduces training visibility, uptake, and sustainability	Memory, attention, Environmental context → Capability, Opportunity	Type: System-level support and evaluation; Mode: Dashboards, outreach strategies, embedded evaluation; Content: Progress tracking, regional communication, QA, and feedback loops	Enhances visibility, accountability, and alignment with system goals; supports continuous improvement

^a^ Based on the Theoretical Domains Framework (TDF) and the Capability, Opportunity, Motivation and Behavior (COM-B) model [8,37].

**Table 3:** Logic model for the digital health capability initiative (Step 3).

Inputs	Activities	Outputs	Outcomes
Funding and strategic leadership	Develop skills-based training grounded in RRR care contexts and aligned with national frameworks	Number of modules developed and delivered	Short-term: Increased awareness and understanding of digital health among RRR health workers and students; improved self-efficacy and foundational digital skills
Subject matter experts, educators, and program staff	Implement self-assessment tools, scenario-based learning, and microcredentials/CPD recognition	Participants by region and profession	
Technological infrastructure and platforms	Establish virtual communities of practice, mentoring networks, and peer learning sessions	Completion, credentialing, and engagement metrics	Medium-term: Consistent application of digital tools in practice, education, and service delivery; improved alignment with local care models; enhanced collaboration through shared platforms
Access to RRR health workforce and organizations	Content designed with end-users and modules tailored for workforce segments	Peer learning sessions or communities of practices established	
Purpose built tools and stakeholder engagement	Track uptake and learner progress via dashboards and feedback loops	Tailored resources produced and usage tracked	Long-term: A digitally confident, future-ready RRR workforce; improved service delivery and workforce satisfaction; reduced digital inequities; strengthened and more resilient health systems
Governance, evaluation, and quality assurance systems	Conduct regional communication campaigns and QA processes	Changes in confidence/capability pre/post training	

## Data Availability

The original contributions presented in this study are included in the article.

## Abbreviations

The following abbreviations are used in this manuscript:

COM-B	Capability, Opportunity, Motivation and Behavior model
DHI	Digital Health Innovation
NARDHC	Northern Australian Regional Digital Health Collaborative
RRR	Rural, Regional, and Remote
TDF	Theoretical Domains Framework
